# Does dietary total antioxidant capacity relate to oxidative stress levels in water immersion during labor? A case-control study

**DOI:** 10.1590/1806-9282.20230996

**Published:** 2024-03-15

**Authors:** Tuğba Küçükkasap Cömert, Seval Yılmaz Ergani, Meltem Uğurlu, Funda Akpınar

**Affiliations:** 1 University of Health Sciences, Gülhane Faculty of Health Sciences, Department of Nutrition and Dietetics – Ankara, Turkey.; 2 University of Health Sciences, Etlik Zübeyde Hanım Training and Research Hospital – Ankara, Turkey.; 3 University of Health Sciences, Gülhane Faculty of Health Sciences, Department of Midwifery – Ankara, Turkey.

**Keywords:** Labor, Antioxidant, Oxidative stress

## Abstract

**OBJECTIVE:**

The aim of this study was to investigate the effect of water immersion during the first stage of labor on maternal and neonatal oxidative stress and the association between serum and dietary total antioxidant capacity.

**METHODS:**

Women were divided into two groups: those immersed in water during the first stage of labor (n=30) and those who had conventional birth (n=33). Total oxidative stress and total antioxidant status levels were examined in antepartum and postpartum maternal serum and neonatal cord blood samples. Dietary total antioxidant capacity was determined by the food frequency questionnaire.

**RESULTS:**

Vitamin C and dietary total antioxidant capacity consumption were found to be higher in the water immersion group (106.92 mg/day and 18.94 mmol/gün, respectively) than the conventional birth group (92.69 mg/day and 15.99 mmol/gün, respectively) (p<0.05). Women immersed in water during the first stage of labor had lower total oxidative stress levels in antepartum and postpartum maternal serum and neonatal cord blood samples than those who had conventional birth (5.43±2.42 mmol/L and 5.59±3.35 mmol/L vs. 8.58±5.53 mmol/L and 12.68±16.58 mmol/L; p<0.05). Dietary total antioxidant capacity was found to be negatively correlated with total oxidative stress levels in antepartum and postpartum maternal serum and neonatal cord blood samples (p=0.012, p=0.047, p=0.035, and p<0.05).

**CONCLUSION:**

Women immersed in water during the first stage of labor had lower total oxidative stress levels in their postnatal maternal serum and neonatal cord blood samples and dietary total antioxidant capacity was also a factor associated with low total oxidative stress levels.

## INTRODUCTION

Pregnancy is a physiological process that increases tissue oxygen and metabolic demands. Increased oxygen demand leads to increased production of free oxygen radicals, leading to increased oxidative stress and lipid peroxidation in pregnant women compared to nonpregnant women^1^. The balance between oxidative and antioxidant systems plays a role in maintaining normal metabolic processes and preventing negative outcomes^2^.

Water immersion during labor (WIDL) is a nonpharmacological conjugate of epidural analgesia. It is becoming a popular choice in contemporary obstetrics because it moves the birthing women from a passive role compliant of authority to an active participant in the whole event, giving a sense of achievement and satisfaction. Both the Royal College of Obstetricians and Gynecologists and the American College of Nurse–Midwives support water immersion in a healthy term "uncomplicated pregnancies"^3^. The 2018 Cochrane review states moderate- to low-quality evidence concerning water immersion during the first stage of labor on the mode of birth (spontaneous, instrumental and cesarean section) and no evidence for adverse neonatal outcomes^4^.

The literature has investigated immersion practices during labor in terms of their effects on labor pain, anxiety, duration of delivery, and the newborn^5-7^. Also, studies have investigated the level of oxidative stress associated with immersion during labor and has been shown to be associated with lower oxidative stress levels. However, they have examined only postpartum values and attributed any differences in oxidative stress between groups to immersion during labor^8,9^.

The present study investigated antepartum, postpartum, and neonatal TOS and TAS and evaluated dietary TAC of maternal diet. We hypothesized that WIDL is associated with lower TOS levels than CB and dietary TAC is a contributing factor to this situation.

## METHODS

### Participants

The study included pregnant women who met the following inclusion criteria: 18–35 years of age; primigravid; term pregnancy (37–41 weeks); no abnormal laboratory findings; no comorbidities; nonsmokers; spontaneous onset of labor; and women with singleton pregnancies, with newborns in vertex presentations and an estimated fetal weight of 2500–4000 g. Pregnant women were excluded if they presented with a ruptured membrane, had a body mass index (BMI) of ≥30 kg/m^2^, or needed labor augmentation.

Sample size was calculated using Student's t-test with 0.80 power at a significant level of 0.05 as described by Sert et al.^8^. The calculation showed that samples should comprise a minimum of 60 pregnant individuals (30 per group) and their newborns.

Pregnant women who met the inclusion criteria were randomly divided into two groups: those immersed in water during the first stage of labor (n=30) (WIDL) and those who were on land during all stages of labor (n=33) (CB).

### Data collection

Maternal and neonatal demographic and obstetric data were extracted from medical records, and data related to mothers’ frequency of food intake in the antenatal period were collected via face-to-face interviews.

### Water immersion conditions

Mothers in the WIDL group had their labors monitored in bathtubs, as described by Ibanoglu et al^9^.

### Biochemical analyses

Total oxidative stress and TAS levels were analyzed using a commercial enzyme-linked immunosorbent assay kit (Relassay, Turkey)^10,11^. The oxidative stress index (OSI) was calculated as the ratio of the TOS level to the TAS level according to the following formula: TOS (μmol H_2_O_2_ equivalent/L)/TAS (μmol Trolox equivalent/L). The OSI is an objective indicator of the balance between TOS and TAS levels^12^.

### Dietary analyses

To determine the daily nutrient intake (energy, macronutrient, and micronutrient intake), a semiquantitative food frequency questionnaire (FFQ) was used and analyzed via the Bebis version 7.2 software (Ebispro, Stuttgart, Germany)^13^. The dietary TAC of the participants was calculated using the antioxidant food database created by Carlsen et al.^14^ based on the ferric-reducing antioxidant power assay.

### Ethical approval

Approval from the ethics committee to conduct this study was obtained from the Clinical Research Ethics Committee of the Etlik Zubeyde Hanim Women's Health Training and Research Hospital (no. 2020/129, dated 09/09/2020). The study was conducted in accordance with the Declaration of Helsinki and followed the ethical standards of the country of origin.

### Statistical analyses

Statistical analysis was performed using the Statistical Package for the Social Sciences (SPSS) version 22.0 for Mac (SPSS Inc., Chicago, IL), and the data distribution was evaluated using the Kolmogorov-Smirnov test and found to be nonnormally distributed. The Mann-Whitney U test and multiple linear regression analysis were used. The level of significance was set at p<0.05.

## RESULTS

There was no difference between the groups in pregnant women's mean age; gestational week; body mass index (BMI); cervical dilatation at the time of admission; estimated fetal weight; duration of the first stage of labor; and newborns’ Apgar scores, birth weights, and neonatal intensive care status. The groups had similar sociodemographic and obstetric characteristics.

Women in the WIDL group had a higher intake of vitamin C (106.92±15.88 mg/day) and dietary TAC than women in the CB group (93.69±16.53 mg/day) (p<0.05) (Table 1).

**Table 1:** Daily energy and nutrient intake of the water immersion during labor and conventional birth groups.

Variable	WIDL (n=30)	CB (n=33)	p
Energy (kcal/day)	1976.81±306.78	1980.33±386.95	0.863
CHO (g/day)	189.95±54.32	182.57±56.98	0.995
Protein (g/day)	75.38±16.52	78.97±17.69	0.457
Fat (g/day)	98.71±20.04	98.78±22.58	0.929
Fiber (g/day)	16.49±5.14	15.51±5.85	0.401
Vitamin A (μg/day)	837.14±600.46	820.24±580.69	0.815
Vitamin E (mg/day)	8.97±3.72	9.25±4.08	0.945
Vitamin B6 (mg/day)	0.78±0.27	0.86±0.30	0.227
Vitamin B12 (μg/day)	3.10±1.93	3.29±2.03	0.751
Folate (μg/day)	230.92±82.26	224.84±80.98	0.809
Calcium (mg/day)	545.56±197.59	541.41±20.61	0.929
Iron (mg/day)	6.71±2.45	7.21±2.69	0.530
Zinc (mg/day)	7.51±2.43	7.68±2.56	0.793
Vitamin C (mg/day)	106.92±15.88	92.69±16.53	0.045*
Dietary TAC (mmol/day)	18.94±5.78	15.99±4.13	0.048*

Dietary TAC: dietary total antioxidant status.

* p<0.05.

Women who had CB had higher postpartum maternal serum TOS levels (8.58±5.53 μmol/L) and neonatal cord blood TOS levels (12.68±16.58 μmol/L) than those who were immersed in water during the first stage of labor (5.43±2.42 μmol/L, 5.59±3.35 μmol/L, and 0.02±0.01, respectively) (p<0.05) (Table 2).

**Table 2:** Biochemical findings in antepartum and postpartum maternal serum samples as well as neonatal cord blood samples.

Variable		WIDL (n=30)	CB (n=33)	p-value
Antepartum maternal serum				
	TOS (μmol/L)	7.82±8.51	6.19±3.98	0.577
	TAS (mmol/L)	2.15±0.21	1.91±0.31	0.121
	OSI	0.66±0.36	0.38±0.16	0.690
Postpartum maternal serum				
	TOS (μmol/L)	5.43±2.42	8.58±5.53	**0.047** *
	TAS (mmol/L)	2.15±0.37	2.04±0.17	0.308
	OSI	0.61±0.59	0.34±0.18	0.074
Neonatal cord blood				
	TOS (μmol/L)	5.59±3.35	12.68±16.58	**0.033** *
	TAS (mmol/L)	2.36±0.41	2.25±0.34	0.121
	OSI	0.61±0.59	0.42±0.33	0.074

TOS: total oxidant status; TAS: total antioxidant status; OSI: oxidative stress index.

* p<0.05.

In the WIDL group, dietary TAC was negatively correlated with antepartum (p=0.000) and postpartum (p=0.014) maternal serum and neonatal cord blood (p=0.001) TOS levels; morever, it was negatively correlated with antepartum (p=0.000) and postpartum (p=0.030) maternal serum OSI levels (p<0.05). Also, dietary TAC was positively correlated with antepartum (p=0.015) and neonatal cord blood (p=0.029) TAS levels (Table 3).

**Table 3:** Multiple linear regression analysis of the effect of dietary total antioxidant capacity on antepartum and postpartum maternal serum and neonatal cord blood samples of total oxidant status, total antioxidant status, and oxidative stress index levels based on birth status.

Variable	Birth status	Beta	t	p	95% confidence interval	
Antepartum maternal serum						
TOS (μmol/L)	WIDL	-0.493	-4.452	0.000*	-0.490	-0.180
	CB	-0.430	-2.743	0.010*	-0.778	-0.113
TAS (μmol/L)	WIDL	0.470	2.604	0.015*	2.614	22.199
	CB	0.444	2.579	0.115	1.213	10.510
OSI	WIDL	-0.561	-4.092	0.000*	-10.552	-31.855
	CB	-0.512	-3.382	0.002*	-5.170	-20.992
Postpartum maternal serum						
TOS (μmol/L)	WIDL	-0.333	-2.626	0.014*	-1.418	-0.173
	CB	-0.091	-0.375	0.711	-0.439	0.303
TAS (μmol/L)	WIDL	0.159	1.138	0.265	14.311	4.111
	CB	0.264	1.179	0.248	8.019	2.156
OSI	WIDL	-0.351	-2.317	0.030*	-2.994	-0.170
	CB	-0.208	-1.437	0.163	-1.495	8.443
Neonatal cord blood						
TOS (μmol/L)	WIDL	-0.456	-3.589	0.001*	-1.236	-0.336
	CB	-0.277	-1.140	0.263	-0.193	0.055
TAS (μmol/L)	WIDL	0.397	2.315	0.029*	0.883	14.851
	CB	0.013	0.058	0.954	4.286	4.534
OSI	WIDL	-0.198	-1.309	0.202	-1.092	-4.921
	CB	-0.362	-0.2173	0.038*	-0.262	-8.635

TOS: total oxidant status, TAS: total antioxidant status, OSI: oxidative stress index.

* p<0.05.

## DISCUSSION

In the current study, we found that postpartum maternal serum and neonatal cord blood TOS levels were found to be lower in the WIDL group than the CB group, and dietary TAC was correlated with antepartum and postpartum maternal serum and neonatal cord blood TAS levels.

Few studies in the literature have investigated the association between immersion during labor and oxidative stress^8,9,15^. From these studies, Sert et al.^8^ investigated the serum levels of disulfide, disulfide/total thiol ratio, native thiol, total thiol, and albumin and Ischemia-modified albumin levels in neonatal cord blood. Ibanoglu et al.^9^ examined the association between WIDL and oxidative stress based on the myeloperoxidase levels in cord blood samples. Assessment of the level of oxidative stress, measuring different oxidant and antioxidant molecules separately, is not recommended because it may cause overlapping and imprecise values as well as high costs; instead, TOS and TAS are advised to be more reliable, sensitive, and stable measures^10,11^. The method of measuring serum TAS and TOS levels in this study has high linearity, and the results are highly reproducible^11^. Also, Uzunlar et al.^15^ used TAS and TOS levels as measures of oxidative and antioxidative stress in relation to immersion and examined these values only in cord blood samples postbirth; they attributed the difference in oxidative stress between the groups to immersion during labor. Our study, unlike the three studies mentioned above, determined antepartum serum TOS and TAS levels and found no difference between the groups. Thus, the present study clearly demonstrated that immersion was the only factor that resulted in low postpartum TOS levels in pregnant women of the WIDL group.

Diet has been the most important contributing factor for the regulation of oxidative stress levels^16^. Maternal dietary TAC (17.32±4.71 mmol/day) of women included in this study was found to be higher than those reported in a study from Brazil that evaluated the dietary TAC of 733 pregnant women (4.3 mmol/day)^17^ but were similar to those reported in another study from Spain (17 mmol/day)^18^. The similarity of the data in the present study to that in the study conducted in Spain can be explained by the Mediterranean diet in both countries. The Mediterranean diet, owing to its abundance of fruits, vegetables, and oilseeds, is high in antioxidants^19^.

It has been reported that dietary antioxidants, such as vitamin C, vitamin E, beta-carotene, and flavonoids, might reduce oxidative stress^20^. In our study results, vitamin C consumption was determined to be higher in the water immersion group than the CB group. This contributes to higher dietary TAC and lower TOS levels determined in the water immersion group.

Instead of analyzing each nutrient with antioxidant properties separately, we preferred to determine the dietary TAC, an indicator of the cumulative ability of diet antioxidants^20^, effect on serum TOS and TAS levels. In addition, our study results showed that there was a relationship between dietary TAC and serum TOS and TAS levels in the WIDL group. In the CB group, a relationship (between dietary TAC and TOS levels) was shown only in the antepartum period. It is thought that this may be related to higher dietary TAC in the water immersion group. Consumption of the recommended amount for dietary TAC has not been reported, and our findings suggest that there may be a threshold for interaction with serum TAS and TOS levels.

## CONCLUSION

This is the first study in which serum oxidative stress levels were determined in prenatal, postnatal, and neonatal cord blood, and their relationship with serum TAS and dietary TAC was evaluated in WIDL.

In this study and other studies, it has been shown that WIDL results in low serum TOS levels. However, in previous studies, dietary TAC, which highly affects serum TOS levels, has not been evaluated. According to our results, dietary TAC also contributes significantly to the provision of low TOS levels.

In conclusion, considering the relationship between dietary TAC and serum TOS levels, an increase in dietary antioxidant intake in the maternal diet can reduce both postpartum maternal serum and cord blood TOS levels.
